# Stability of 10 Beta-Lactam Antibiotics in Human Plasma at Different Storage Conditions

**DOI:** 10.1097/FTD.0000000000001100

**Published:** 2023-08-21

**Authors:** Soma Bahmany, Tim M.J. Ewoldt, Alan Abdulla, Birgit C.P. Koch

**Affiliations:** Departments of * Hospital Pharmacy, † Intensive Care Medicine, Erasmus University Medical Center, Rotterdam, The Netherlands;; ‡ CATOR: Center for Antimicrobial Treatment Optimization Rotterdam, The Netherlands.

**Keywords:** beta-lactam antibiotics, stability, degradation, chromatography, mass spectrometry

## Abstract

**Background:**

Recently, several studies have assessed the effects of therapeutic drug monitoring of frequently prescribed beta-lactam antibiotics, for which they were quantified in human plasma samples. Beta-lactams are considered unstable, leading to extra challenges in quantification. Therefore, to ensure sample stability and minimize sample degradation before analysis, stability studies are crucial. This study investigated the stability of 10 frequently used beta-lactam antibiotics in human plasma at relevant storage conditions for clinical use.

**Methods:**

Amoxicillin, benzylpenicillin, cefotaxime, ceftazidime, ceftriaxone, cefuroxime, flucloxacillin, imipenem, meropenem, and piperacillin were analyzed using ultraperformance convergence chromatography tandem mass spectrometry and liquid chromatography tandem mass spectrometry. Their short-term and long-term stabilities were investigated by measuring quality control samples at low and high concentrations against freshly prepared calibration standards. Measured concentrations at each time point were compared with the concentrations at T = 0. Antibiotics were considered stable if recovery results were between 85% and 115%.

**Results:**

Short-term stability results indicated ceftriaxone, cefuroxime, and meropenem to be stable up to 24 hours at room temperature. All evaluated antibiotics, except imipenem, were stable on ice in a cool box for 24 hours. Amoxicillin, benzylpenicillin, and piperacillin were stable for 24 hours at 4–6°C. Cefotaxime, ceftazidime, cefuroxime, and meropenem were stable at 4–6°C up to 72 hours. Ceftriaxone and flucloxacillin were stable for 1 week at 4–6°C. Long-term stability results showed that all antibiotics were stable up to 1 year at −80°C, except imipenem and piperacillin, which were stable for 6 months at −80°C.

**Conclusions:**

Plasma samples for amoxicillin, benzylpenicillin, cefotaxime, ceftazidime, flucloxacillin, and piperacillin may be stored for a maximum of 24 hours in a cool box. Refrigeration is suitable for plasma samples of amoxicillin, benzylpenicillin, meropenem, and piperacillin for up to 24 hours and cefotaxime, ceftriaxone, ceftazidime and cefuroxime for 72 hours. Plasma samples for imipenem should be frozen directly at −80°C. For long-term storage, plasma samples can be stored at −80°C for a maximum of 6 months for imipenem and piperacillin and 12 months for all other evaluated antibiotics.

## Abstract 2

Supplemental Digital Content is Available in the Text.

## BACKGROUND

Beta-lactam antibiotics (penicillins, cephalosporins, monobactams, and carbapenems) are among the most important and commonly prescribed antibiotics. Attainment of therapeutic levels of these antibiotics is essential in effectively treating severe bacterial infections.^1^ However, in certain populations with altered pharmacokinetics, such as patients with obesity, elderly patients, children, or patients with burns or critical illnesses, attainment of therapeutic levels of beta-lactam antibiotics is suboptimal. To optimize dosing strategies for these populations, the application of therapeutic drug monitoring (TDM) by measuring drug concentrations in plasma samples has gained momentum in recent years. Several recent studies were performed to assess the effects of the application of TDM for frequently prescribed beta-lactam antibiotics.^2^ TDM is used to optimize pharmacological target attainment and thereby leads to improved patient outcomes.^3^ Whether the impact of increased therapeutic level attainment as a result of TDM has positive effects on the clinical outcomes of critically ill patients is yet to be confirmed.^1,4,5^

For the quantification of antibiotic levels in clinical practice, an accurate, precise, and selective method is required. An additional challenge of beta-lactam antibiotics TDM is that these antibiotics are unstable compounds.^6,7^ Their instability is due to the presence of a beta-lactam ring in their chemical structure, which can break up readily and is susceptible to hydrolysis. Understanding the stability profile of these compounds is crucial for the accuracy of the measured concentrations. Degradation of these antibiotics in samples may cause misrepresentation of actual concentrations at the time the sample was taken, which could result in incorrect dosing. Due to the relative novelty and moderate availability of beta-lactam antibiotic analytical methods, samples are often not analyzed on a daily basis or are transported to a specialized laboratory for analysis. Cooled transport or cooled storage before analysis are not standard and accrue additional costs. Furthermore, if a sample requires reanalysis or additional analysis after regular storage, the stability profile must be available. Therefore, stability studies are essential to avoid sample degradation before analysis.

Previously, several stability studies were performed, which observed instability of beta-lactam antibiotics.^8–11^ However, most of these studies evaluated a limited number beta-lactam antibiotics at limited storage conditions and time points. The aim of this study was to investigate the stability of 10 frequently used beta-lactam antibiotics in human plasma at 4 different storage conditions for up to 1 year to ensure correct sample handling processes in clinical practice for TDM.

## MATERIALS AND METHODS

### Methodology

The stability of amoxicillin, benzylpenicillin, cefotaxime, ceftazidime, ceftriaxone, cefuroxime, flucloxacillin, imipenem, meropenem, and piperacillin in human plasma was investigated for 1 year at suitable and relevant time points and at 4 different storage conditions; at room temperature (RT 20°C), on ice in a cool box (−10 to +2°C), refrigerator (4–6°C), and freezer (−80°C). The temperature in the cool box was measured by a temperature logger (EL-USB-2 temperature data logger). Chemical structures of all evaluated antibiotics are presented in Table 1, and the evaluated time points are presented in Table 2. Relevant storage conditions for TDM were investigated. For example, before transport to the laboratory, study samples may stay at RT at the clinical departments for up to 24 hours. However, most study samples are transferred immediately into a cool box or refrigerator before transportation to the laboratory. For this reason, stability in the cool box and refrigerator were also investigated. After collecting the study samples at the laboratory, samples are centrifuged for 6 minutes at 1811*g*. Thereafter, plasma samples are transferred into a cryo tube and stored at −80°C before analysis. Unfortunately, in practice, plasma samples may not always be frozen immediately after collecting them at the laboratory. Therefore, stability of samples in the refrigerator was also evaluated for up to 1 week.^12^ The study was performed according to the relevant European Medicines Agency and Food and Drug Administration guidelines. Quality control (QC) samples were prepared at 2 concentrations, low (L) and high (H), using citrate-anticoagulated plasma. The stability of these antibiotics was investigated by measuring QC low and QC high samples in quadruplicate against freshly prepared calibration standards. Analytical quantification was performed using liquid chromatography (LC) and ultraperformance convergence chromatography (UPC^2^) coupled by tandem mass spectrometry (MS/MS). Measured concentrations at each time point were compared with the concentrations at T = 0. Compounds were considered to be stable if recovery percentages were between 85% and 115%.

**TABLE 1.** Chemical Structures of Evaluated Antibiotics

Analyte	Chemical Structure
Amoxicillin	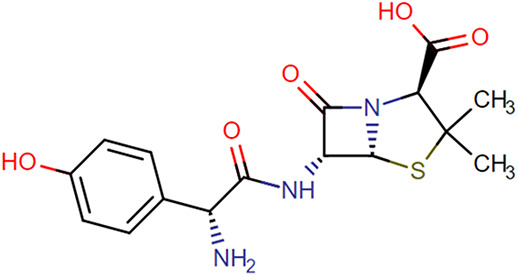
Benzylpenicillin	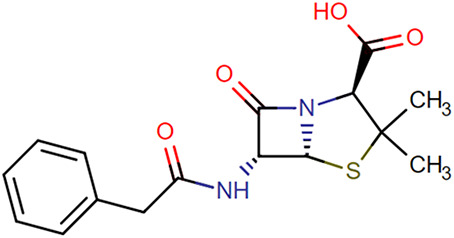
Cefotaxime	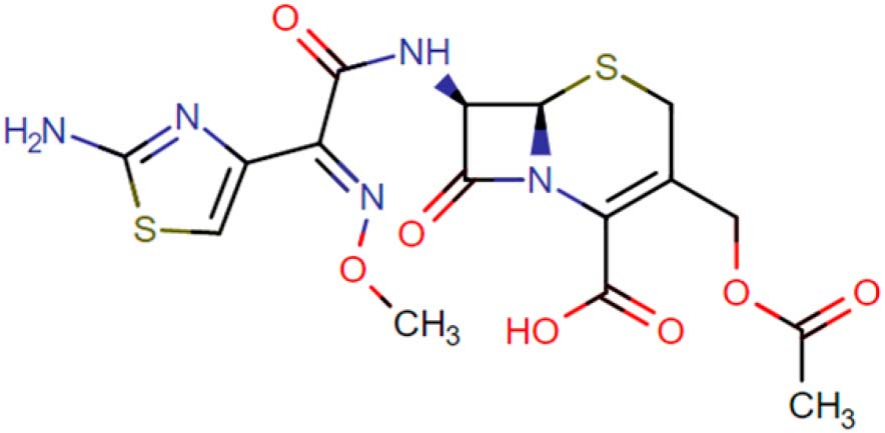
Ceftazidime	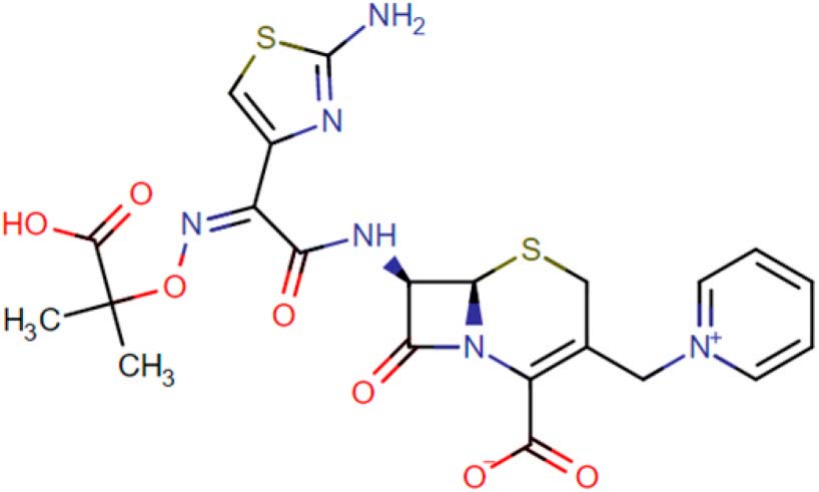
Ceftriaxone	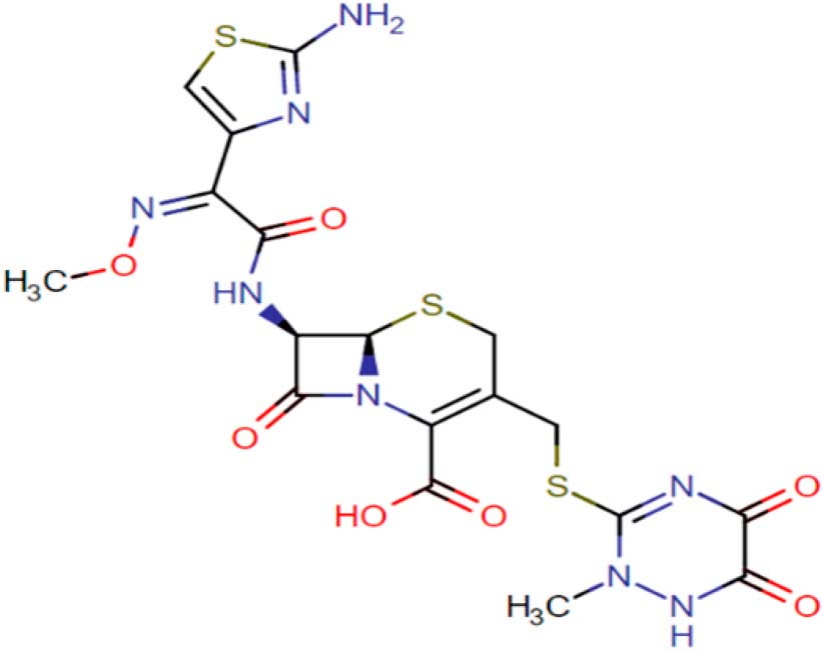
Cefuroxime	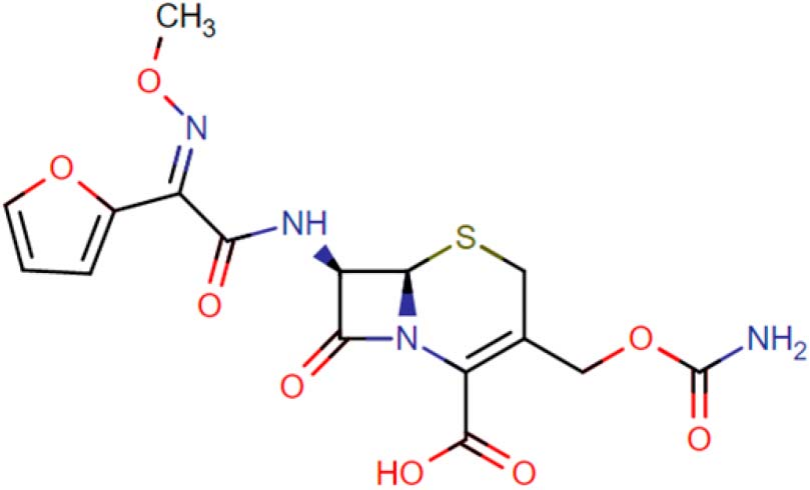
Flucloxacillin	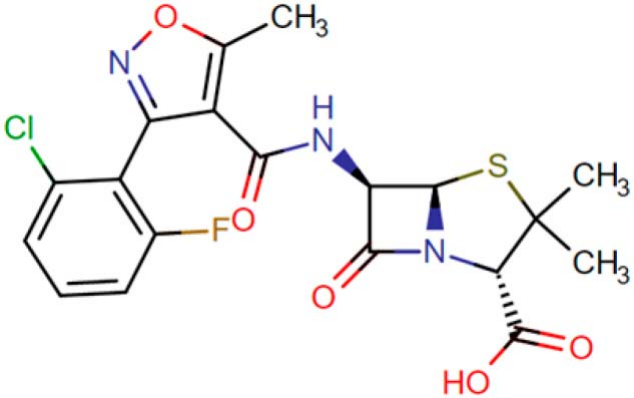
Imipenem	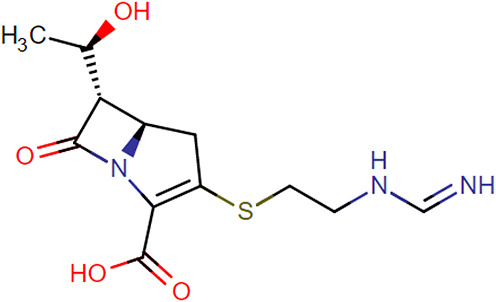
Meropenem	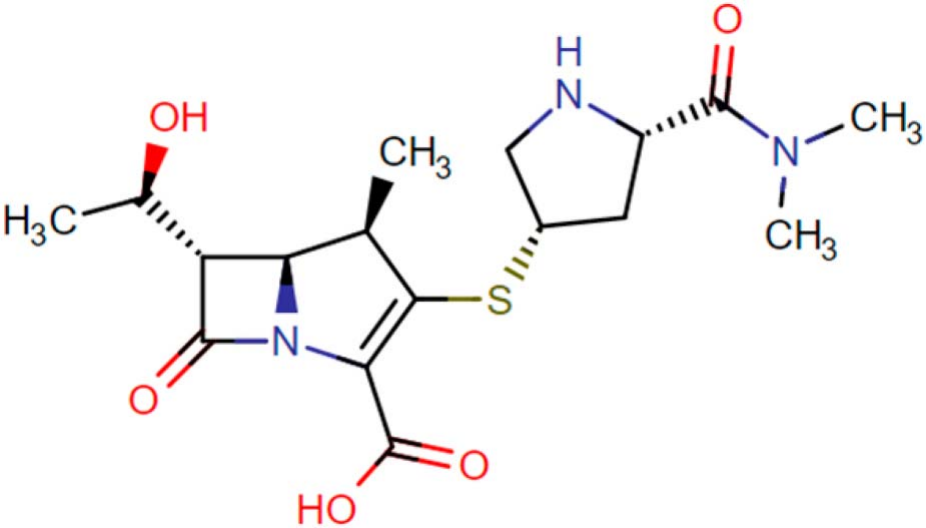
Piperacillin	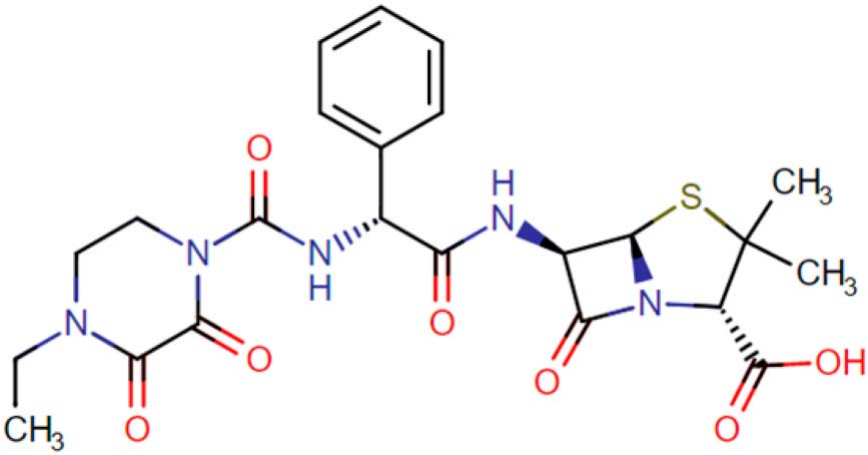

**TABLE 2.** Evaluated Time Points for Short-Term And Long-Term Stability

Time-points in hours (h) and months (mo)	RT	On Ice*	4–6°C	−80°C
24 h	Yes	Yes	Yes	ND
72 h	ND	ND	Yes	ND
168 h	ND	ND	Yes	Yes
1 mo	ND	ND	ND	Yes
2 mo	ND	ND	ND	Yes
3 mo	ND	ND	ND	Yes
6 mo	ND	ND	ND	Yes
12 mo	ND	ND	ND	Yes

* In a cool box (−10 to 2°C).

RT (20°C).

## MATERIALS

### Chemicals

Cefotaxime sodium salt and cefazoline sodium salt were obtained from Sigma-Aldrich Chemie (Zwijndrecht, The Netherlands), and ammonium formate was obtained from Fluka Honeywell Research Chemicals (Bucharest, Romania). Amoxicillin and benzylpenicillin were obtained from SelleckChem (Houston, TX) and flucloxacillin from Toronto Research Chemicals (North York, ON, Canada). Piperacillin, cefuroxime, ceftazidime, imipenem, and meropenem trihydrate were obtained from Santa Cruz Biotechnology (Dallas, TX). Ceftriaxone and meropenem-d6 were purchased from Cayman Chemical (Ann Arbor, MI). LC-MS methanol (99%) was purchased from Biosolve (Valkenswaard, The Netherlands). Ultrapure water was prepared in-house using a MilliPore Advantage A10 System (Millipore, Bedford, MA). Drug-free human plasma was obtained from Sanquin Bloodbank (Amsterdam, The Netherlands).

### Stock Solutions, Calibration Standards, Quality Control Samples, and Internal Standard

Stock solutions of each compound were prepared. Stock solutions for benzylpenicillin, ceftazidime, ceftriaxone, and imipenem were prepared in MilliQ water. Amoxicillin, flucloxacillin, piperacillin, cefazoline, cefotaxime, cefuroxime, meropenem-d6, and meropenem were solubilized in methanol. All stock solutions were directly used for the preparation of the quality controls at high and low concentrations. Blank human plasma was spiked with the stock solutions for the preparation of the QC samples. QC samples were prepared separately for carbapenems, cephalosporins, penicillins, and ceftriaxone. Ceftriaxone belongs to the cephalosporins but was analyzed using a different quantification method. Concentrations of all QC samples are presented in Table 3. A 50-µL aliquot of each QC sample was transferred into 1.5-mL safe-lock Eppendorf tubes and stored at 4 different storage conditions.

**TABLE 3.** Mean Concentrations of Each Quality Control Sample at T = 0

Analyte	QC L (mg/L)	QC H (mg/L)
Amoxicillin	4	15
Benzylpenicillin	20	80
Cefotaxime	10	40
Ceftazidime	16	65
Ceftriaxone	4	15
Cefuroxime	12	50
Flucloxacillin	20	80
Imipenem	6	25
Meropenem	10	25
Piperacillin	20	85

### Instrumentation

Amoxicillin, benzylpenicillin, flucloxacillin, piperacillin, cefotaxime, cefuroxime, ceftazidime, imipenem, and meropenem samples were analyzed using a Waters Acquity UPC^2^-MS/MS system, which was connected to a Waters TQ-S micro triple quadrupole mass spectrometer (Waters, Milford, MA). The Acquity UPC^2^ system consisted of an Acquity binary solvent manager (chromatographic pump), sample manager (autosampler), column manager (column oven), convergence manager, and isocratic solvent manager (a specific pump to create a separate flow from eluent B to the mass spectrometer). Data acquisition and analysis were conducted using Masslynx V4.1 (Waters). Ceftriaxone was analyzed using a Dionex Ultimate UPLC system, which was connected to a Thermo Triple Stage Quadrupole Vantage system consisting of a triple quadrupole mass spectrometer. The UPLC system consisted of an Ultimate 3000 RS UPLC-pump, an autosampler, and a column oven. Xcalibur 2.1 and Chromeleon 6.80 (all Thermo Scientific, Waltham, MA) were used for data acquisition.

### Chromatographic and MS/MS Settings

Two different quantification methods were used during this stability study. Both methods are validated according to the relevant FDA guidelines and EMA guidelines.^12,13^ Validation results are summarized in **Supplemental Digital Content 1** (see **Table S1**, http://links.lww.com/TDM/A666).

### Carbapenems, Cephalosporins, and Penicillins

These compounds were analyzed according to a previously published quantification method.^14^ Chromatographic separation was performed using a Waters UPC^2^ Torus Diol 2.1 × 100 mm column with a 1.7-µm particle size. Mobile phase A consisted of liquid CO_2_, and mobile phase B consisted of 0.01mol/L ammonium formate (pH 6.5) in LC-MS methanol (5% vol/vol). The gradient elution program started with 30% of eluent B and increased to 55% of eluent B after 1 minute. Eluent B was kept at 55% from 1 to 4 minutes, and an equilibration step was applied at starting conditions from 4 to 5 minutes. Column temperature was set at 55°C. The flow rate was 0.75 mL/min, and the makeup flow rate (which only consisted of eluent B) was 0.2 mL/min. Injection volume was set at 20 µL. The total runtime was 5.0 minutes per sample.

### Ceftriaxone

Chromatographic separation of ceftriaxone was achieved on a Waters Acquity UPLC BEH C18 2.1 × 50 mm column with a 1.7-µm particle size. A gradient elution program was applied using 0.002 mol/L of ammonium formate + 0.1% formic acid (FA) in Milli-Q water as mobile phase A and 0.002 mol/L ammonium formate + 0.1% FA as mobile phase B with a flow rate of 0.5 mL/min. The gradient started with 10% of eluent B and was increased to 100% B after 1 minute and hold at 100% until 2.8 minutes. The gradient was reset to starting conditions (10% B) from 2.9 to 4.5 minutes to equilibrate for the next injection. Column temperature was set at 50°C. Injection volume was set at 1 µL. Total run time per sample was 4.5 minutes. Mass spectrometry settings of all compounds are presented in Table 4.

**TABLE 4.** Acquisition Parameters Used in the MS/MS Method for the Selected Antibiotics

Analyte	ESI Mode	Parent Ion (m/z)	Daughter Ion (m/z)	Collision Energy (V)	Dwell Time (ms)
Amoxicillin	+	365.99	113.94	22	50
Benzylpenicillin	+	334.92	160.04	8	50
Cefotaxime	+	455.93	125.12	46	50
Ceftazidime	+	546.97	468.03	10	50
Ceftriaxone	+	555.20	125.05	50	70
Cefuroxime	+	441.98	364.01	8	50
Flucloxacillin	+	453.88	160.02	16	50
Imipenem	+	299.87	97.89	26	50
Meropenem	+	383.98	68.05	32	50
Piperacillin	+	518.08	143.06	20	50

### Sample Preparations

#### Carbapenems, Cephalosporins, and Penicillins

A 50-µL aliquot of each QC sample was used for sample preparation, to which 800 µL of the internal standard working solution (2 mg/L of meropenem-d6 in methanol) was added and vortexed for 10 seconds. After vortexing, samples were centrifuged for 5 minutes at 1811*g*. Thereafter, 50 µL of the supernatant was transferred to an amber 2-mL autosampler vial, and 950 µL of eluent B was added and vortexed for 10 seconds.

#### Ceftriaxone

A 50-µL aliquot of each QC sample was used for sample preparation, to which 400 µL of the internal standard working solution (5 mg/L of cefazoline in methanol) was added and vortexed for 10 seconds. After vortexing, samples were centrifuged for 5 minutes at 1811*g*. Then, 200 µL of the supernatant was transferred to an amber autosampler vial with a 0.3-mL insert.

## RESULTS

**FIGURE 1.**
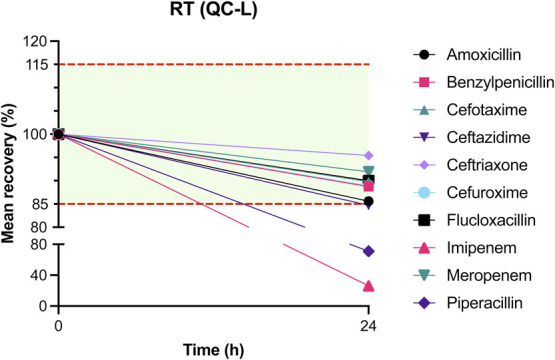
Mean recovery of QC L at RT. The green area represents the acceptable stability range. Measurements outside the red dotted line were considered unstable.

**FIGURE 2.**
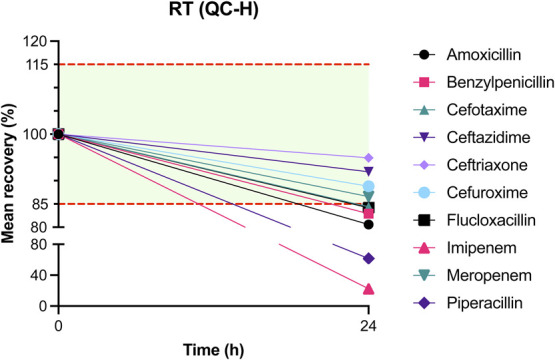
Mean recovery of QC H at RT. The green area represents the acceptable stability range. Measurements outside the red dotted line were considered unstable.

**FIGURE 3.**
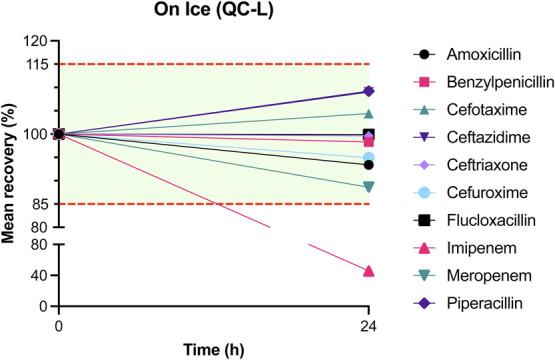
Mean recovery of QC L on ice. The green area represents the acceptable stability range. Measurements outside the red dotted line were considered unstable.

**FIGURE 4.**
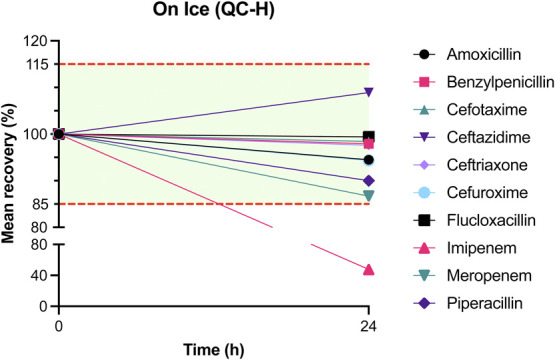
Mean recovery of QC H on ice. The green area represents the acceptable stability range. Measurements outside the red dotted line were considered unstable.

**FIGURE 5.**
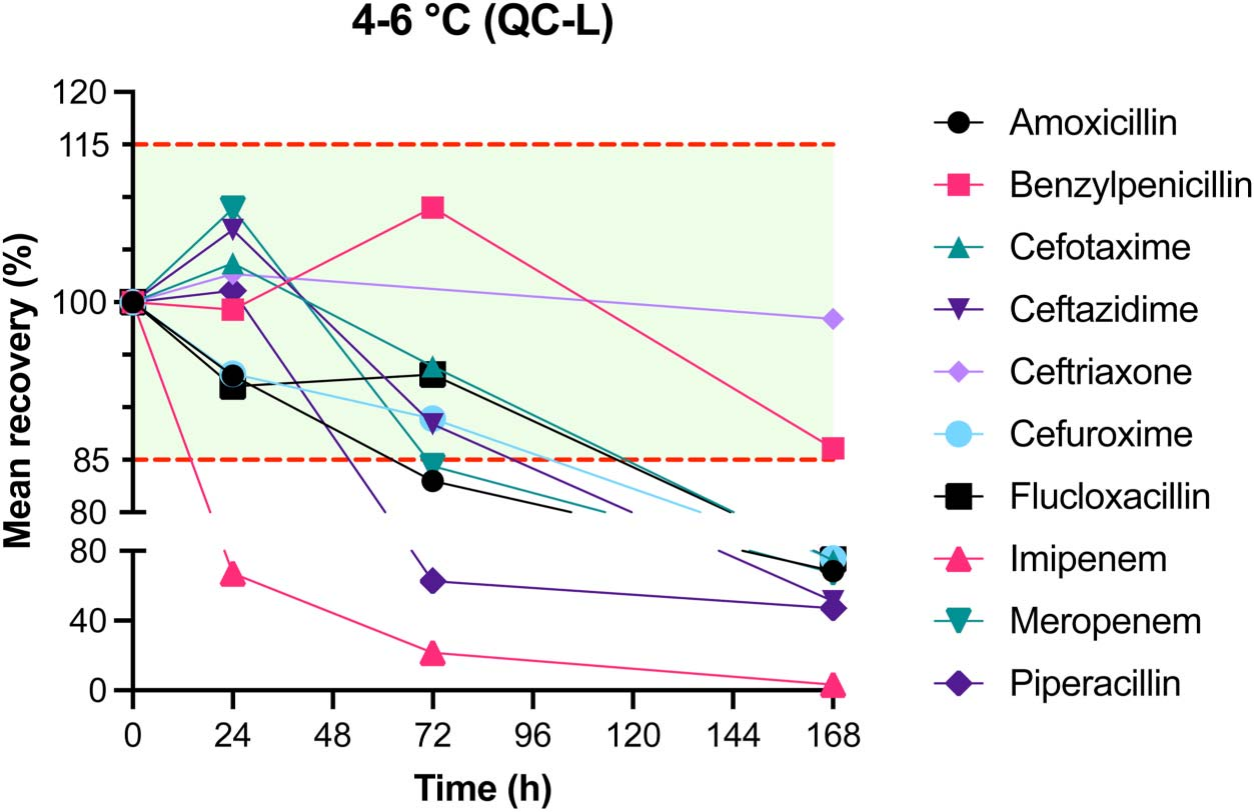
Mean recovery of QC L in the refrigerator. The green area represents the acceptable stability range. Measurements outside the red dotted line were considered unstable.

**FIGURE 6.**
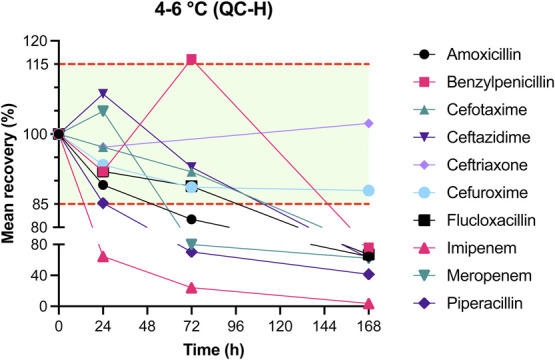
Mean recovery of QC H in the refrigerator. The green area represents the acceptable stability range. Measurements outside the red dotted line were considered unstable.

**FIGURE 7.**
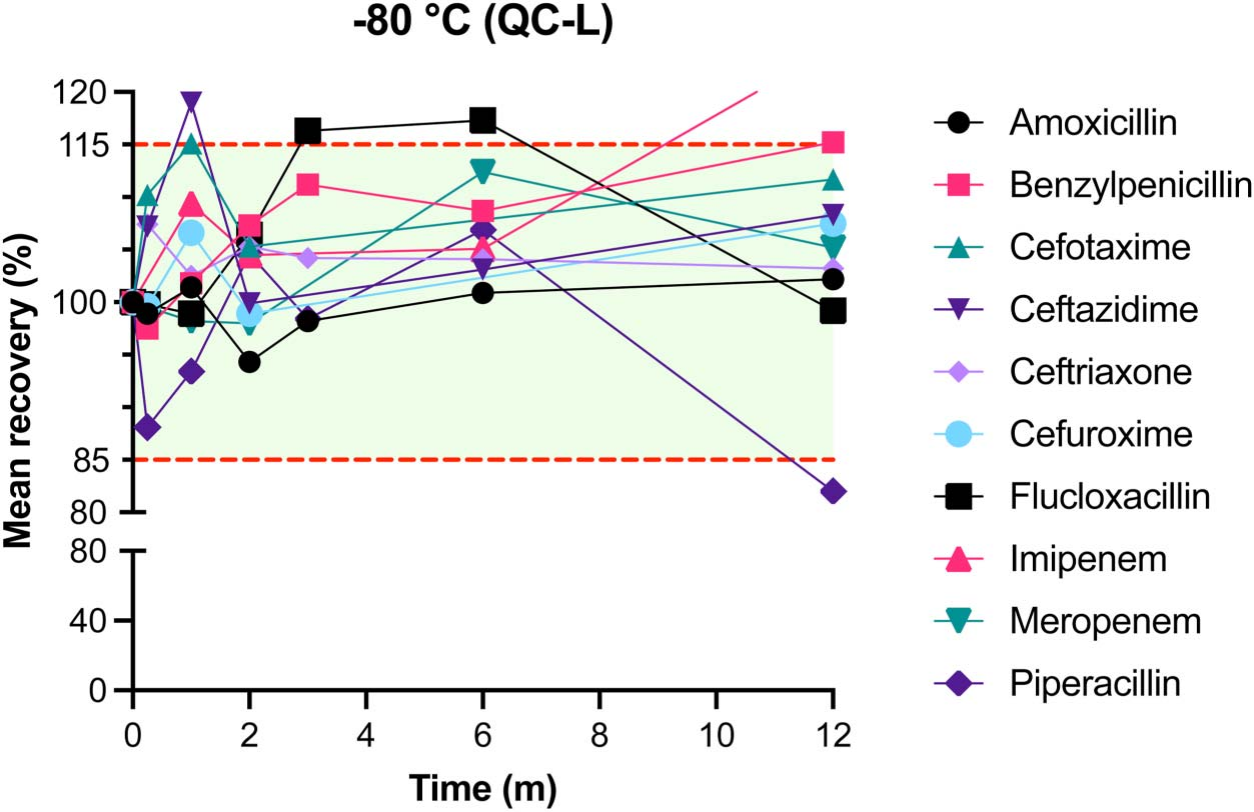
Mean recovery of QC L in the freezer (−80°C). The green area represents the acceptable stability range. Measurements outside the red dotted line were considered unstable.

**FIGURE 8.**
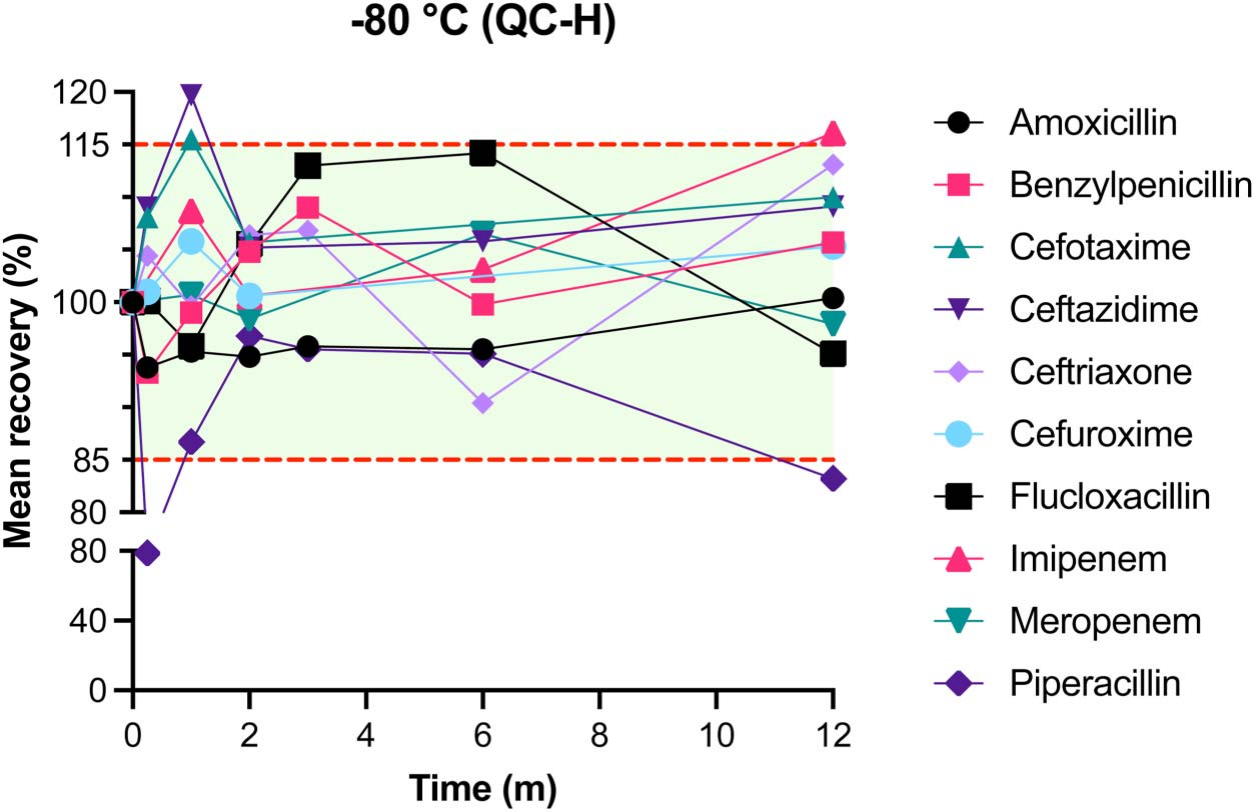
Mean recovery of QC H in the freezer (−80°C). The green area represents the acceptable stability range. Measurements outside the red dotted line were considered unstable.

The results of mean recovery percentages at evaluated time points are presented in Figures 1–8. Mean recoveries for each time point are presented in **Supplemental Digital Content 2** (see **Tables S2 and S3**, http://links.lww.com/TDM/A667, http://links.lww.com/TDM/A671). Antibiotics were reported as stable when the mean recovery results were within 85% and 115%.

Results of the short-term stability study showed that ceftriaxone, cefuroxime, and meropenem are stable at RT for 24 hours. During storage in a cool box and refrigerator, all antibiotics, with the exception of imipenem, were considered to be stable for 24 hours. Cefotaxime, ceftazidime, cefuroxime, and meropenem were found to be stable for 72 hours in the refrigerator. Ceftriaxone and flucloxacillin were stable for 1 week in the refrigerator.

Results of the long-term stability study showed that amoxicillin, benzylpenicillin, cefotaxime, ceftazidime, ceftriaxone, cefuroxime, flucloxacillin, and meropenem were found to be stable for 1 year at −80°C. Piperacillin degraded by more than 15% after 12 months of storage at −80°C. Positive recoveries of imipenem were observed after 12 months of storage. More positive recoveries were observed for cefotaxime, ceftazidime, and flucloxacillin at the selected midrange time points. Mean recoveries at T = 1 month for cefotaxime and ceftazidime were 115.1% and 119.0% for QC L and 115.5% and 119.7% for QC H. Mean recoveries for QC L of flucloxacillin were 116.3% and 117.3% at T = 6 months.

## DISCUSSION

We have found that ceftriaxone, cefuroxime, and meropenem were stable for 24 hours when stored at RT. All evaluated beta-lactam antibiotics, with the exception of imipenem, were stable on ice or in the refrigerator for at least 24 hours. Results of the long-term stability study showed that all evaluated antibiotics were stable at −80°C for 12 months with the exception of imipenem and piperacillin, which were stable for 6 months at −80°C.

Our findings for the short-term stability study of ceftriaxone, cefuroxime, and meropenem at RT contradicted other stability studies of meropenem,^8–11^ which showed faster degradation. The stabilities observed for amoxicillin, benzyl penicillin, ceftazidime, cefuroxime, and piperacillin under refrigeration conditions are in line with the stability results of Van Vooren et al^15^ Stability study results for ceftazidime, meropenem, and piperacillin under refrigeration conditions reported by Mortensen et al^8^ is comparable to our results. However, our stability results for meropenem and flucloxacillin are contrary to the results of studies by D'Cunha et al^10^ and van Vooren et al,^15^ which showed faster degradation under refrigeration conditions. These contradictions could be caused by different anticoagulants or matrix compositions used in the studies.

Remarkably, rapid degradation of imipenem was observed compared with that of meropenem, which is notable because both antibiotics have largely similar molecular structures. A possible explanation for this observation might be the missing methyl group on the first carbon of the carbapenem moiety of imipenem, which confers lower stability. These findings indicate that imipenem samples should be transported on ice, and samples should be immediately stored in the freezer (−80°C). In addition, we observed faster degradation of penicillins compared with that of cephalosporins. This phenomenon could be caused by the unstable, highly strained, and reactive beta-lactam amide bond in the chemical structures of the penicillins.^7^ Other factors that contribute toward instability of penicillins are the beta-lactam carbonyl group and acyl side chain that undergo neighboring group participation, which leads to opening of the beta-lactam ring.

All antibiotics, with the exception of imipenem and piperacillin, were found to be stable for 1 year when stored at −80°C. Pinder et al^11^ evaluated the long-term stability of ceftazidime, meropenem, piperacillin, and flucloxacillin after 9 and 13 months of storage at −80°C. Their results for ceftazidime, flucloxacillin, and piperacillin are comparable to our results. In contradiction to our results for meropenem, Pinder et al^11^ observed more than 15% degradation after 1 year of storage. Our findings for the recovery of meropenem after 12 months of storage at −80°C were 105% and 98% for QC L and QC H, respectively. The possible causes of this difference are not clear.

This stability study has a few limitations. First, positive recoveries were obtained for cefotaxime, ceftazidime, and flucloxacillin at a few middle time points. Besides that, some data were missing at the middle time points. However, because the results of earlier and later evaluated time points are within the acceptance criteria, and the positive recoveries were small and therefore negligible, it may be scientifically justifiable to make a final decision based on the latest time point.^16^ Second, despite our extensive stability study, the effects of freeze–thaw cycles were not investigated. For this reason, further studies to evaluate the freeze–thaw effect for each compound are warranted. Furthermore, in practice, plasma is not always immediately isolated from the whole blood sample. Therefore, there is scope for further evaluation of antibiotic stability in whole blood samples under the different storage conditions. Finally, in case of reanalysis of study samples for amoxicillin, benzylpenicillin, cefotaxime, cefuroxime, ceftazidime, flucloxacillin, and meropenem, it may be useful to evaluate the long-term stability in the freezer (−80°C) at additional 15-, 18-, and 24-month time points.

## CONCLUSIONS

Based on our findings, we recommend that plasma samples for amoxicillin, benzylpenicillin, cefotaxime, ceftazidime, flucloxacillin, and piperacillin are stored immediately in a cool box or refrigerator. Plasma samples for amoxicillin, benzylpenicillin, meropenem, and piperacillin could be stored in a cool box or refrigerator for a maximum of 24 hours. Plasma samples for cefotaxime, ceftriaxone, ceftazidime, and cefuroxime could be refrigerated for a maximum of 72 hours. Plasma samples for imipenem should be immediately frozen at −80°C.

For long-term storage, we recommend that plasma samples are stored at −80°C for a maximum of 6 months for imipenem and piperacillin and a maximum of 12 months for all other evaluated antibiotics. Calibration standards and quality control samples for clinical routine analysis could be prepared in advance and stored at −80°C, which is advantageous and convenient for daily routine analysis.

## Supplementary Material
