# Supplementary material for: Stability of 10 Beta-Lactam Antibiotics in Human Plasma at Different Storage Conditions
Source: Ther Drug Monit. 2023 Aug 21;45(5):606–15. doi: 10.1097/FTD.0000000000001100 (PMC10497202; doi:10.1097/FTD.0000000000001100)
Supplement: Supplementary file 1 [file tdm-45-606-s001.docx]

| **Analyte** | **QC** | **Conc.**  **(mg/L)** | **Accuracy**  CV (%) | **Intra-day imprecision**  CV (%) | **Inter-day imprecision**  CV (%) |
| --- | --- | --- | --- | --- | --- |
| Amoxicillin | Low  Medium  High | 5.0  10.1  20.2 | -5.6  -11.1  -1.2 | 5.3  5.4  5.7 | 7.2  9.1  4.7 |
| Benzylpenicillin | Low  Medium  High | 16.9  33.9  67.7 | -1.4  -0.3  9.5 | 8.5  10.9  5.7 | 5.0  7.6  8.5 |
| Cefotaxime | Low  Medium  High | 9.3  18.6  37.2 | 2.8  3.0  4.7 | 3.1  2.7  3.8 | 5.8  6.8  8.0 |
| Ceftazidime | Low  Medium  High | 8.0  15.9  31.9 | 8.0  8.4  9.2 | 3.2  3.5  4.9 | 8.4  6.3  6.9 |
| Ceftriaxone | Low  Medium  High | 4.0  8.0  16.0 | -5.2  -2.6  -2.0 | 5.5  2.3  3.4 | 6.2  5.1  6.6 |
| Cefuroxime | Low  Medium  High | 7.5  15.1  30.2 | -0.3  -1.6  0.9 | 6.3  3.0  6.5 | 8.1  7.0  7.6 |
| Flucloxacillin | Low  Medium  High | 21.3  42.7  85.4 | 0.8  -1.5  0.5 | 6.9  5.8  5.6 | 7.2  7.6  7.9 |
| Imipenem | Low  Medium  High | 10.8  21.6  43.3 | 2.7  -4.1  -6.2 | 2.6  2.4  4.7 | 4.2  5.0  6.0 |
| Meropenem | Low  Medium  High | 9.8  19.6  39.2 | 4.3  -0.5  0.4 | 2.7  2.4  3.8 | 3.0  4.8  5.4 |
| Piperacillin | Low  Medium  High | 20.8  41.5  83.1 | -5.6  -6.0  10.1 | 6.7  5.2  7.3 | 7.7  6.7  6.0 |

**SUPPLEMENTAL DATA**

**Table S1**: Summary of validation data
