# Supplementary material for: Stability of 10 Beta-Lactam Antibiotics in Human Plasma at Different Storage Conditions
Source: Ther Drug Monit. 2023 Aug 21;45(5):606–15. doi: 10.1097/FTD.0000000000001100 (PMC10497202; doi:10.1097/FTD.0000000000001100)
Supplement: Supplementary file 2 [file tdm-45-606-s002.docx]

**SUPPLEMENTAL DATA**

**Table S2**: Mean recovery (%) of QC samples stored at room temperature (20 °C), in a cool box on ice (-10 to - 2 °C), and refrigerator (4–6 °C) compared to T = 0

| Analyte | QC sample | RT  T = 24h | On Ice  T = 24h | 4-6 °C  T = 24h | 4-6 °C  T = 72h | 4-6 °C  T = 168h |
| --- | --- | --- | --- | --- | --- | --- |
| Amoxicillin | L  H | 85.6  80.6 | 93.4  94.5 | 93.0  89.1 | 83.0  81.7 | 68.3  64.3 |
| Benzylpenicillin | L  H | 88.7  82.9 | 98.3  97.9 | 99.3  92.0 | 109.0  116.1 | 86.1  76.0 |
| Cefotaxime | L  H | 89.8  84.4 | 104.4  98.4 | 103.7  97.2 | 93.9  91.9 | 74.6  76.4 |
| Ceftazidime | L  H | 84.7  91.9 | 109.0  108.9 | 106.9  108.6 | 88.4  92.9 | 51.1  62.3 |
| Ceftriaxone | L  H | 95.4  94.9 | 99.6  97.6 | 102.7  97.2 |  | 98.4  102.3 |
| Cefuroxime | L  H | 88.9  88.8 | 94.9  94.3 | 93.2  93.4 | 88.9  88.6 | 75.6  87.9 |
| Flucloxacillin | L  H | 90.0  84.2 | 99.9  99.4 | 93.3  92.0 | 93.1  88.8 | 74.9  66.9 |
| Imipenem | L  H | 26.2  22.4 | 45.9  47.7 | 67.2  64.6 | 21.7  24.1 | 3.3  3.7 |
| Meropenem | L  H | 91.9  86.6 | 88.6  86.7 | 108.9  104.9 | 84.4  79.9 | 67.4  62.0 |
| Piperacillin | L  H | 70.9  61.7 | 109.2  90.0 | 101.1  85.2 | 62.6  70.3 | 47.2  41.3 |
